# Perceptions and Intentions of Nursing Students Regarding Digital Health: Cross-Sectional Study

**DOI:** 10.2196/77051

**Published:** 2026-03-05

**Authors:** Alexandre Castonguay, Sandrine Hegg-Deloye, Guy Paré, Faustin Armel Etindele Sosso

**Affiliations:** 1 Faculté des sciences infirmières, Université de Montréal, 2375 chemin de la Côte-Sainte-Catherine, Montreal, QC, H3T 1A8, Canada, 1 514-343-6437 ext 13751; 2 Département de technologie de l’information, HEC Montréal, Montreal, QC, Canada; 3 Research Chair in Digital Health, HEC Montréal, Montreal, QC, Canada; 4 Université de Montréal, Montreal, QC, Canada

**Keywords:** health IT, digital health education, eHealth competencies, technology adoption in nursing, informatics competency, nursing education, health information systems

## Abstract

**Background:**

The integration of digital health technologies (DHTs) in clinical practice is accelerating, creating a need for nursing students to develop digital competencies aligned with professional expectations. In Quebec, curricular reforms aim to enhance digital health literacy, but data are limited on students’ preparedness.

**Objective:**

This study aimed to assess nursing students’ perceptions, self-reported competencies, and willingness to engage with DHTs across different academic years.

**Methods:**

A cross-sectional descriptive survey assessing self-reported digital health competencies, attitudes, perceived training coverage, and intentions was conducted using an online questionnaire administered through Qualtrics. Participants (N=136) were recruited from 3 cohorts: first-year (group 1; n=58, 42.6%), second-year (group 2; n=55, 40.4%), and third-year (group 3; n=23, 16.9%) nursing students. Data were analyzed using descriptive statistics and ANOVAs, with post hoc analyses performed via SPSS.

**Results:**

Significant differences were observed among cohorts concerning digital competencies and access to digital tools. Compared with first-year students (group 1), third-year students (group 3) showed higher proficiency with electronic medical records (group 3: mean 3.29, SD 1.31; group 1: mean 2.59, SD 1.32; *P*=.01), virtual reality (group 3: mean 4.53, SD 1.11; group 1: mean 2.90, SD 1.44; *P*<.001), and clinical databases (group 3: mean 4.59, SD 1.00; group 1: mean 3.21, SD 1.55; *P*<.001). Despite positive attitudes toward DHTs across all groups, training coverage for most digital tools was perceived as low, with the highest levels reported for clinical databases (mean 2.97, SD 1.1). This underscored a substantial gap between institutional expectations and actual digital training across all cohorts.

**Conclusions:**

This study highlights critical gaps in digital health training among nursing students, emphasizing the need for targeted curricular reforms such as the one currently underway at the Université de Montréal. These efforts represent a promising opportunity to better align educational content with the evolving demands of health care systems. Today, preparing students in digital competencies is no longer just advantageous but may soon become essential for the next generation of nurses to navigate and lead within technology-driven care environments.

## Introduction

### Macro-Level Context: Digital Shift in Health Care

The digital transformation of health care systems is rapidly redefining how care is delivered, documented, and managed worldwide. Across clinical environments, digital health technologies (DHTs)—including electronic medical records (EMRs), virtual care platforms, and artificial intelligence—are increasingly deployed to enhance care coordination, improve efficiency, and respond to growing service demands [1,2]. In Quebec, this shift is occurring amid a persistent nursing shortage that places additional pressure on health care institutions and personnel [3]. Strategic investments in digital infrastructure, such as those outlined in the province’s 2022 to 2025 technological modernization plan and the federal Pan-Canadian Health Data Strategy, have positioned digital tools as key enablers of more responsive, equitable, and sustainable care delivery [4,5].

### Professional Standards and Competencies

To ensure that nurses are prepared to operate within these digitized environments, regulatory and educational bodies have issued guidelines calling for the integration of informatics into nursing practice. The Ordre des infirmières et infirmiers du Québec (OIIQ) emphasizes the importance of digital competencies for safe, ethical, and efficient care across both in-person and virtual settings [6,7]. At the national level, the Canadian Association of Schools of Nursing (CASN) has updated its National Nursing Education Framework to include explicit expectations for digital health literacy among nursing graduates [8]. Together, these standards promote the incorporation of information and communications technologies into nursing curricula as both a professional requirement and a pedagogical priority.

### Educational Challenges in Nursing Programs

Despite these mandates, gaps remain in the actual delivery of digital health training across Canadian nursing programs. While students often demonstrate general digital literacy, studies show that they are underprepared to use clinical information systems, navigate electronic records, or interpret data generated by digital tools in real-world practice [9-11]. Furthermore, many programs offer inconsistent or superficial exposure to health ITs (HITs), contributing to low confidence and proficiency among graduates [11]. These limitations risk widening the gap between institutional expectations and actual clinical readiness, particularly as the pace of digitalization continues to accelerate. Previous studies indicate that, although nursing students tend to have a positive attitude toward digital health, their clinical readiness remains limited due to inconsistent exposure to health information systems. Competency frameworks such as those from the CASN and OIIQ underscore the need for structured, progressive integration of digital training into nursing education [7,8]. These limitations contribute to low confidence and perceived readiness among graduates entering technology-rich clinical environments.

### Study Rationale in the Quebec Context

Although several studies have examined nurses’ perceptions of digital health tools, few have focused on nursing students in Quebec, where the educational structure, regulatory environment, and pace of curricular reform differ from those of other Canadian provinces [12]. Given recent efforts at the Université de Montréal (UdeM) to revise its undergraduate nursing program to better align with CASN and OIIQ guidelines, it is crucial to understand students’ current perceptions, competencies, and intentions regarding DHTs. To our knowledge, this is the first study in Quebec to use a cohort-comparative design to examine digital health preparedness among nursing students. Grounded in the technology acceptance model and the OIIQ digital competency framework, this study aimed to identify year-based differences in perceptions and training gaps, thereby informing targeted curricular reforms to support Quebec’s digital health transformation [12,13].

This study was conceptually inspired by the behavioral framework by Paré et al [14] examining medical students’ intention to integrate DHTs, which combines elements from the theory of interpersonal behavior by Triandis [15] and the technology acceptance model [16]. The survey content was further adapted to the nursing context using insights from the 2020 National Survey of Canadian Nurses on the use and impact of DHTs in practice. The final instrument was reviewed and validated in collaboration with experienced nursing professors from the Faculty of Nursing (Faculté des sciences infirmières in French; FSI) at UdeM to ensure relevance and clarity for nursing students.

### Research Objectives

This study had the following objectives:

- Explore the perceptions and intentions of nursing students regarding their knowledge, experiences, and training related to HITs and their beliefs, attitudes, and intentions concerning the use of HITs in their future professional practice
- Compare these perceptions and competencies based on students’ year of study to identify potential differences across cohorts
- Provide recommendations for improving HIT-related education in response to the identified needs

## Methods

### Design, Setting, and Participants

This study used a cross-sectional descriptive design. It was conducted at the FSI at UdeM in May 2023. The target population included 1274 students enrolled in the FSI at UdeM. A target sample size of 300 was chosen based on recommended minimum thresholds for descriptive surveys in large student populations [17] to ensure reasonable representation as no formal power calculation was required for this exploratory design. Eligible participants were all students currently enrolled in an undergraduate nursing program who provided informed consent.

It is important to note that participant distribution across academic years was uneven. This reflects actual enrollment sizes within the program during the data collection period.

### Ethical Considerations

This study was reviewed and approved by UdeM’s Research Ethics Committee in Sciences and Health (2023-4300) and was supported by the FSI. All participants provided informed consent electronically before starting the survey and could withdraw at any time without consequence. Data were collected anonymously to ensure participant privacy and confidentiality. No financial or other compensation was provided for participation.

### Recruitment

UdeM was selected as the research site due to its status as a major higher education institution in Quebec and the diversity of its undergraduate nursing student population. Recruitment was conducted electronically through the FSI, which emailed an initial invitation and one reminder to all eligible students via their institutional addresses. Interested participants accessed the study by clicking a secure link leading to the consent form and the online questionnaire. Participation was voluntary and anonymous. Although IP addresses were not collected to ensure confidentiality, the use of institutional email distribution and limited reminders minimized the risk of multiple entries.

### Data Collection

Data were collected through an online self-administered questionnaire designed specifically for this study administered via Qualtrics (Qualtrics International Inc), a platform designed for web-based research that offers basic descriptive statistics tools while ensuring secure data handling.

The questionnaire took approximately 15 minutes to complete and included an open-ended question at the end, allowing participants to share additional comments. Although this open-ended question was included at the end of the questionnaire to capture additional student perspectives, these responses were not systematically analyzed in this study. An exploratory review of these responses was conducted to inductively identify recurring themes and enrich the interpretation of the quantitative findings by reflecting participants’ lived experiences and unprompted perspectives.

The survey was developed to operationalize key constructs from the conceptual framework described above. Specific items were adapted from the study by Paré et al [14] and the 2020 National Survey of Canadian Nurses to align with the nursing context and relevant DHTs. The draft instrument was then reviewed and refined in collaboration with experienced nursing faculty at UdeM to ensure content validity and clarity. The final version included 15 items (Table S1 in Multimedia Appendix 1) and is available in Multimedia Appendix 2.

### Variables

This study assessed nursing students’ perceptions, competencies, and training needs related to HITs across 4 main categories of variables.

#### Sociodemographic and Academic Variables

Sociodemographic and academic variables included the year and type of academic program; the participants’ age, sex, and gender; languages of interaction; daily use of digital devices; previous professional experience in the health care sector; and current or intended professional setting after graduation.

#### HIT Competencies and Training

Using Likert-type scales, participants assessed their level of proficiency with various technologies (1=“none,” 2=“very low,” 3=“low,” 4=“moderate,” 5=“high,” and 6=“very high”), the extent to which HITs were covered in their nursing education program (1=“not covered at all,” 2=“poorly covered,” 3=“moderately covered,” 4=“well covered,” and 5=“very well covered”), and the level of expertise they believed was necessary for clinical practice (1=“no training needed,” 2=“basic training,” 3=“functional or intermediate training,” and 4=“expert or specialized training).

#### Perceived Impacts of HITs

Participants were asked to rate the perceived impact of DHTs on key aspects of the nursing profession, such as quality of care and work life. The question was phrased as follows: “Do you believe that the use of digital health technologies has, or will have, an impact on the following dimensions of the nursing profession?” Responses were collected on a Likert scale (1=“very negative impact,” 2=“negative impact,” 3=“neutral impact,” 4=“positive impact,” and 5=“very positive impact”). An open-ended question followed, allowing respondents to elaborate or provide additional comments.

### Statistical Analysis

Students were grouped into 3 categories based on their year of study: first year of the integrated diploma of college studies–bachelor’s degree (*diplôme d'études collégiales–baccalauréat* in French; DEC-BAC) and regular bachelor’s degree (*baccalauréat* in French; BAC) programs (group 1), second year of the DEC-BAC and BAC programs (group 2), and third year of the DEC-BAC and BAC programs (group 3). All Likert scale variables were treated as continuous variables and analyzed using means and SDs.

For categorical variables, frequencies and percentages were reported for each group as well as for the total sample. Group comparisons were conducted using exact *P* values from chi-square tests. For continuous variables, valid sample sizes, means, and SDs were presented. Group means for Likert scale variables were compared using one-way ANOVA followed by Tukey post hoc tests where appropriate.

An exception was made for the variable “training coverage—scheduling software,” where the Welch correction for unequal variances was applied and post hoc comparisons were conducted using Games-Howell tests. All statistical analyses were performed using the SPSS software (version 28; IBM Corp), with a significance level set at 5%.

## Results

### Sociodemographic and Academic Variables

#### Overview

A total of 173 students accessed the questionnaire. The response rate for the item identifying participants’ academic year within the nursing program was 78.6% (136/173). After grouping, group 1 comprised 42.6% (58/136) first-year students, group 2 comprised 40.4% (55/136) second-year students, and group 3 comprised 16.9% (23/136) third-year students.

#### Participant Profile

Across all groups, more than 67% (92/136) of participants were aged between 18 and 25 years, with group 3 standing out at 80% (19/23) in this age range (*P*=.03). This group also reported significantly higher access to a laptop (*P*=.02). Use of digital tools for specific activities was also significantly greater among groups 2 and 3 (*P*=.01). Additionally, a higher proportion of group 3 participants reported working in the provincial public health care sector (*P*=.003; Table S2 in Multimedia Appendix 1).

### HIT Competencies and Training

#### HIT Proficiency Levels

Proficiency levels across all work-related HITs were generally rated from low to moderate. Group 2 stood out in terms of proficiency with EMRs (*P*=.01), whereas group 3 demonstrated higher proficiency with virtual reality (*P*=.001) and clinical databases (*P*=.001; Table S3 in Multimedia Appendix 1).

#### Training Coverage by Group

Regardless of the specific HIT in question, coverage within the curriculum was generally perceived as very limited to minimal. Group 1 reported greater coverage in the use of scheduling software (*P*=.007), whereas group 3 reported more extensive coverage in database training (*P*=.004; Table S4 in Multimedia Appendix 1).

#### Required Expertise for Professional Use of HITs

Participants generally believed that most HITs required little to no specific expertise for professional use. An exception was noted for group 2, which reported a significantly higher need for basic or intermediate training in the use of health care robots (*P*=.02; Table S5 in Multimedia Appendix 1).

### Perceived Impacts of HITs in the Nursing Profession

Perceptions of the impact of HITs on the nursing profession were consistent across all groups. Most students believed that DHTs have a positive or very positive impact on key aspects such as quality of care, therapeutic relationships, and productivity (Table S6 in Multimedia Appendix 1).

### Narrative Comments

Of the 136 respondents, 31 (22.8%) provided written responses to the open-ended question. Most comments reflected key patterns observed in the quantitative results, particularly regarding the perceived lack of formal training in DHTs. Several students reported learning to use digital tools only during clinical placements, consistent with the limited curriculum coverage ratings found across cohorts. One respondent noted that “[w]e only learn to use digital tools during internships,” whereas another remarked that “[a]t university, we have no training on the software we encounter in practice.”

Others described barriers to accessing technologies during clinical placements due to their student status:

As a nurse it’s fine, but as a student, it’s complicated when we don’t have the access.

These comments aligned with cohort-based differences observed in digital tool use and confidence levels.

However, the responses also surfaced dimensions not captured in the structured survey. Several participants mentioned psychosocial or physical impacts of digital tool use, including “less sleep, eye strain, and a more sedentary lifestyle” and “technology sometimes limits social interaction, which affects mental health.” A few students proposed improvements, such as earlier exposure to clinical systems or greater consistency in tools across health care settings:

It would help to learn some of the clinical software in advance.

Please standardize the digital documents, it would simplify things when moving between institutions.

While broadly aligned with the quantitative trends, these narrative comments introduced experiential nuances, such as well-being, autonomy, and implementation barriers, that were not accessible through the Likert scale items alone.

## Discussion

### Principal Findings

Both the OIIQ Comité jeunesse and the OIIQ [6,12] emphasize the importance of access to IT that supports nurses’ clinical decision-making, optimizes care processes, and enhances patient safety, as well as maintaining professional and ethical standards.

Similarly, the CASN, through its National Nursing Education Framework [8], underscores the importance of integrating nursing informatics competencies into academic programs to better prepare future professionals for the increasing technological demands of clinical environments.

However, our findings indicate that students perceive their current training as insufficient. Their self-assessed proficiency with digital tools was limited, and coverage of key HITs was generally perceived as weak without a clear progression across academic years despite students showing an interest in these tools and their potential impact on nursing practice.

These observations suggest a concerning disconnect between the evolving expectations of the health care system and students’ perceived preparedness despite the clear guidelines set forth by both the OIIQ and CASN. This aligns with findings by Kleib et al [11], who identified persistent gaps in digital health education that were exacerbated by pedagogical approaches that often overestimate students’ technological competencies and fail to adequately prepare them for clinical realities.

The narrative comments reinforced this disconnect by providing concrete examples of students’ perceived lack of preparation, particularly in relation to clinical software. The comments also highlighted barriers that were not captured in the structured survey, such as restricted access to digital tools during placements and the personal toll of technology use on well-being. These experiential accounts complement the quantitative findings and emphasize that perceived readiness is shaped not only by curricular content but also by the conditions of access, support, and emotional experience within clinical environments.

The fact that many participants in our study believed that high-level proficiency was unnecessary for the effective use of HITs may reflect a limited understanding of their relevance in nursing practice. Kleib et al [11] emphasize that, while students often demonstrate basic digital literacy, this does not necessarily translate into clinical competence. Consequently, students may underestimate the complexity and significance of digital tools in real-world settings. These findings highlight the pivotal role of nursing educators in bridging this gap by explicitly integrating digital competencies into nursing curricula and grounding them within authentic clinical contexts to foster both awareness and applied proficiency.

Despite these challenges, the surveyed students remained generally optimistic about the role of HITs, with most believing that these technologies will have a positive or even very positive impact on the nursing profession. This optimism reflects a growing awareness of the digital shift in health care and aligns with the strategic directions promoted by the OIIQ and CASN, who advocate for stronger digital health training in nursing education [7,8].

Beyond these overarching findings, notable differences emerged between academic years in terms of access to digital tools, proficiency with specific technologies, and perceived curriculum coverage. This study identified several statistically significant differences in how students from each cohort perceived and interacted with HITs.

The 18- to 25-year age group predominated across all cohorts, particularly in group 3, where it represented 83% (19/23) of the participants. Group 3 students also reported greater access to digital tools, especially laptops, and more advanced use of EMRs, virtual reality, and databases. These findings are consistent with the broader and more in-depth training coverage that this group reported for these tools.

The observed differences in competencies and training exposure likely reflect curricular progression, with third-year students benefiting from more extensive clinical placements, capstone projects, and practical assignments that integrate digital tools. In addition, group 3 students may have greater opportunities for independent learning and part-time work in health care settings, reinforcing their proficiency with EMRs and clinical databases. In contrast, group 1 students are typically at the beginning of their program, focused on foundational theoretical content with limited hands-on exposure to applied digital health systems.

Group 2 students stood out for their more frequent use of digital tools and a higher reported need for advanced competencies in health care robotics. Meanwhile, group 1 students reported better coverage related to scheduling software, likely reflecting academic needs specific to first-year studies.

Taken together, these findings reveal a persistent and concerning gap between current digital health needs and the perceived preparedness of nursing students, echoing the concerns expressed by the OIIQ [7]. They underscore the importance of continuing efforts to integrate digital health competencies into nursing education in a structured and strategic manner.

### Limitations and Future Research

#### Methodological and Contextual Limitations

Of the 1274 targeted students, 136 (10.7%) completed the questionnaire, representing 45.3% (136/300) of the sample size required to ensure statistical representation. While the target of 300 respondents was chosen based on practical guidelines for descriptive surveys, the absence of a formal power calculation means that the study may be underpowered to detect smaller effect sizes or subtle group differences, particularly given the uneven distribution across cohorts.

The unequal sample sizes between academic years may also have limited the statistical power for some comparisons. However, this limitation mirrors real enrollment patterns and was accounted for using appropriate statistical analyses.

Although no data were available to confirm a nonresponse bias, the relatively low participation rate raises the possibility of such a risk. Therefore, it is possible that respondents had characteristics or perceptions that differed from those of nonrespondents, potentially limiting the generalizability of the findings.

Additionally, the self-administered nature of the questionnaire introduces the possibility of self-selection and self-assessment bias. Students who chose to participate may have been more comfortable with technology or more aware of digital health issues, potentially skewing responses toward more favorable or more critical perceptions than would be typical. Moreover, as the study was conducted at a single institution, UdeM, the transferability of the findings to other educational, institutional, or cultural contexts is limited. The uneven distribution of participants across groups, particularly the low number of participants in group 3, further reduces the robustness of statistical comparisons and may have hindered the detection of significant effects in certain analyses.

In addition, while this study included an open-ended question that generated valuable narrative insights, these data were reviewed only through an exploratory, unsystematic process and were not coded or analyzed using a formal qualitative methodology. As such, they cannot be considered representative or exhaustive. Even so, the added value of these responses, particularly in revealing experiential and emotional dimensions absent from the structured survey, suggests that future research would benefit from an intentionally designed mixed methods approach. Incorporating qualitative inquiry from the outset guided by a clear theoretical framework would allow for a more comprehensive understanding of how nursing students engage with digital technologies across educational and clinical contexts.

#### Future Research Directions

The FSI at UdeM is currently in the process of implementing a revised version of its undergraduate program in response to recent recommendations from the CASN that emphasize the importance of developing digital competencies aligned with current clinical demands and realities.

In this context, a pretest-posttest design with independent cohorts comparing students who completed the old curriculum with those trained under the revised program would offer valuable insights into the impact of the reform on digital health literacy, integration, and appropriation. Additionally, consultations with key stakeholders within the faculty, including teaching staff, program directors, educational advisors, and student representatives from each cohort, could provide critical institutional perspectives on the validity of the findings presented in this study. As part of a complementary qualitative component, this triangulated approach would not only contextualize the results but also help assess how curriculum reform may influence digital health teaching practices.

Several additional methodological avenues could be explored to build on this work and deepen understanding of students’ perceptions and competencies regarding HITs. First, replicating the study across multiple nursing schools through a multisite comparative design would help assess the transferability of findings beyond the UdeM context. Second, adopting a mixed methods approach to combine quantitative questionnaires with qualitative interviews could yield a more nuanced understanding of student expectations, perceived barriers, and enabling conditions for meaningful engagement with digital tools. Finally, longitudinal studies conducted throughout the academic journey would allow for documentation of digital competency development over time in relation to different education milestones and ongoing curricular adjustments.

### Conclusions, Implications, and Recommendations

This study reveals a significant gap between institutional expectations and perceptions of digital health training by nursing students, particularly in their proficiency with essential tools such as EMRs and clinical databases. Despite recognizing the value of digital technologies, students reported limited curricular coverage and confidence in using them.

These findings call for targeted reforms in nursing education, including the integration of hands-on training, digital simulations, and interactive modules. To address these gaps more concretely, nursing programs could implement several specific measures: (1) dedicated simulation laboratories focused on EMRs and clinical software; (2) mandatory digital health literacy modules that introduce students to national and provincial informatics frameworks; and (3) interprofessional digital health workshops that mirror real-world collaborative settings involving nurses, physicians, and allied health professionals. These recommendations are further supported by students’ own accounts, which reflect both a desire for earlier, more practical exposure to digital tools and a recognition of the limitations in their current training environment.

The current curriculum revision at UdeM offers a timely opportunity to evaluate the implementation outcomes of such changes. Future studies using a clearly defined pretest-posttest design, with precise cohort tracking to distinguish students enrolled before, during, or after curriculum reforms and collecting detailed data on external exposure to HITs (eg, work settings) could help assess the true impact of these reforms.

Broader research across institutions and through mixed methods or longitudinal approaches will be crucial to strengthening the evidence base literature. In an increasingly technology-driven health care landscape, equipping the next generation of nurses with robust digital competencies is no longer optional and has become a professional imperative.

## Supplementary material

10.2196/77051 Multimedia Appendix 1 Descriptive and comparative tables summarizing participant characteristics, technology proficiency, training coverage, and perceived expertise requirements across student groups.

10.2196/77051 Multimedia Appendix 2 Study questionnaire assessing nursing students’ perceptions, experiences, and attitudes toward digital health technologies.

## Abbreviations

BAC: baccalauréat
CASN: Canadian Association of Schools of Nursing
DEC-BAC: diplôme d'études collégiales–baccalauréat
DHT: digital health technology
EMR: electronic medical record
FSI: Faculty of Nursing
HIT: health information technology
OIIQ: Ordre des infirmières et infirmiers du Québec
UdeM: University of Montreal
