# Correction: Perceptions and Intentions of Nursing Students Regarding Digital Health: Cross-Sectional Study

**DOI:** 10.2196/98552

**Published:** 2026-04-30

**Authors:** Alexandre Castonguay, Sandrine Hegg-Deloye, Guy Paré, Faustin Armel Etindele Sosso

**Affiliations:** 1 Faculté des sciences infirmières, Université de Montréal, 2375 chemin de la Côte-Sainte-Catherine, Montreal, QC, H3T 1A8, Canada, 1 514 343 6437 ext 13751; 2 Département de technologie de l’information, HEC Montréal, Montreal, QC, Canada; 3 Research Chair in Digital Health, HEC Montréal, Montreal, QC, Canada; 4 Université de Montréal, Montreal, QC, Canada

 In “Perceptions and Intentions of Nursing Students Regarding Digital Health: Cross-Sectional Study” [1], the authors made one correction.

The file originally published as Multimedia Appendix 2 has been replaced with the correct questionnaire corresponding to this study, which is attached here as Multimedia Appendix 1.

The correction will appear in the online version of the paper on the JMIR Publications website, together with the publication of this correction notice. Because this was made after submission to PubMed, PubMed Central, and other full-text repositories, the corrected article has also been resubmitted to those repositories.

## Supplementary material

10.2196/98552 Multimedia Appendix 1 Study questionnaire assessing nursing students’ perceptions, experiences, and attitudes toward digital health technologies (revised Multimedia Appendix 2).
