# Supplementary material for: Perceptions and Intentions of Nursing Students Regarding Digital Health: Cross-Sectional Study
Source: JMIR Nurs. 2026 Mar 5;9:e77051. doi: 10.2196/77051 (PMC12978967; doi:10.2196/77051)
Supplement: Multimedia Appendix 1 [file nursing-v9-e77051-s001.docx]

Table 1. Variables and items

| **Model Variable** | **Number of Associated Items** |
| --- | --- |
| Participant Profile | 6 |
| Experience with Health Information Technologies | 2 |
| Beliefs About Digital Health | 2 |
| Attitudes Toward Digital Health | 1 |
| Social Norms | 1 |
| Perceived Behavioral Control | 1 |
| Behavioral Intention | 1 |
| Open-Ended Comments | 1 |

Table 2. Descriptive Statistics by Group and Chi-Square Results

|  | Group 1(n=58) | Group 2, (n=55) | Group 3, (n=23) | Total, (N=136) | P value |
| --- | --- | --- | --- | --- | --- |
| **Female, n (%)** | 48 (82.8) | 51 (92.7) | 21 (91.3) | 120 (88.2) | .24 |
| **Age group. n (%)** | | | | | |
| 18-25 years | 35 (60.3) | 38 (69.1) | 19 (82.6). | 92 (67.6) | **.033** |
| 26-35 years | 7 (12.1) | 10 (18.2) | 2 (8.7 | 19 (13.9) | .075 |
| 36-45 years | 3 (5.2) | 1 (1.8) | 2 (8.7) | 6 (4.4) | .61 |
| Missing data | 13 (22.4) | 6 (10.91) | 0 (0.0) | 19 (13.9) | .001 |
| **Language(s) used in interactions. n (%)** | | | | |  |
| French | 44 (75.9) | 48 (87.3) | 22 (95.7) | 114 (83.8) | .06 |
| English | 23 (39.7) | 26 (47.3) | 20 (87.0) | 69 (50.7) | **<.001** |
| Autre | 2 (3.4) | 2 (3.6) | 2 (8.7) | 6 (4.4) | .74 |
| Missing data | 13 (22.4) | 6 (10.9) | 0 (0.0) | 19 (13.9) | 001 |
| **Daily use of electronic devices. n (%)** | | | | | |
| Smartphone | 45 (77.6) | 46 (83.6) | 22 (95.7) | 113 (83.1) | .14 |
| Tablet | 36 (62.1) | 28 (50.9) | 12 (52.2) | 76 (55.9) | .48 |
| Laptop | 40 (69.0) | 45 (81.8) | 22 (95.7) | 107 (78.7) | **.024** |
| Desktop computer | 7 (12.1) | 9 (16.4) | 7 (30.4) | 23 (16.9) | .13 |
| Other | 2 (3.4) | 1 (1.8) | 1 (4.3) | 4 (2.9) | 1.000 |
| Missing data | 0 (0.0) | 0 (0.0) | 0 (0.0) | 0 (0.0) | 1.000 |
| **Prior professional experience in healthcare. n (%)** | | | | | |
| Yes. with digital tools | 13 (22.4) | 29 (52.7) | 12 (52.2) | 54 (40.0) | **.011** |
| Yes. without digital tools | 11 (18.9) | 4 (7.3) | 5 (21.7) | 20 (14.8) |  |
| No experience | 26 (44.8) | 18 (32.7) | 6 (26.1) | 50 (37.0) |  |
| Missing data | 8 (13.8) | 4 (7.3) | 0 (0.0) | 12 (8.8) | 1.000 |
| **Current or last work setting in healthcare. n (%)** | | | | | |
| Community health sector | 2 (3.4) | 2(3.6) | 1(4.3) | 5 (3.6) | .82 |
| Private health sector | 1 (1.7) | 2 (3.6) | 2 (8.7) | 5 (3.6) | .82 |
| Provincial public health sector | 12 (20.7) | 32 (58.2) | 16 (69.6) | 60 (44.1) | **.003** |
| Federal public health sector | 0 (0.0) | 2 (3.6) | 0 (0.0) | 2 (1.4) | .13 |
| Academic sector | 1 (1.7) | 0 (0.0) | 0 (0.0) | 1 (0.7) | .37 |
| Not worked as a nurse | 26 (44.8) | 9 (16.3) | 3 (13.0) | 38 (27.9) | **<.001** |
| Other | 2 (3.45) | 2 (3.6) | 1 (4.3) | 5 (3.6) | .82 |
| Missing data | 14 (24.1) | 6 (10.9) | 0 (0.0) | 20 (14.0) | **<.001** |
| **Intended future work sector. n (%)** | | | | | |
| Nurse – Private sector | 18 (31.0) | 7 (12.7) | 4 (17.4) | 29 (21.3) | .12 |
| Nurse – Public sector | 22 (37.9) | 35 (63.6) | 16 (69.6) | 73 (53.7) |  |
| Academic field (teaching/research) | 2 (3.4) | 1 (1.8) | 1 (4.3) | 4 (2.9) |  |
| Other sector | 2 (3.4) | 2 (3.6) | 0 (0.0) | 4 (2.9) |  |
| Undecided | 13 (22.4) | 9 (16.4) | 2 (8.7) | 24 (17.6) |  |

Table 3. Proficiency Levels by Group and Mean Comparisons (Mean ± SD)

| Technology, (n) mean (SD) | Group 1 (n=58) | Group 2 (n=55) | Group 3 (n=23) | Total (N=136) | P value |
| --- | --- | --- | --- | --- | --- |
| General technology use | (29) 5.34 ( 0.7) | (34) 5.26 ( 0.9) | (17) 5.47 ( 0.6) | (80) 5.34 ( 0.8) | .68 |
| Specialized websites | (29) 4.00 ( 1.0) | (34) 4.38 ( 0.9) | (17) 4.53 ( 0.9) | (80) 4.28 ( 0.9) | .12 |
| Mobile applications | (29) 2.21 ( 1.6) | (34) 2.47 ( 1.7) | (17) 2.47 ( 1.7) | (80) 2.38 ( 1.7) | .80 |
| Electronic medical records (EMRs) | (29) 2.59 ( 1.3) | (34) 3.59 ( 1.3) | (17) 3.29 ( 1.3) | (80) 3.16 ( 1.3) | **.011** |
| Scheduling software | (29) 3.31 ( 1.5) | (34) 3.59 ( 1.6) | (17) 2.47 ( 1.5) | (80) 3.25 ( 1.6) | .05 |
| Nursing care plan software | (29) 2.52 ( 1.4) | (34) 3.06 ( 1.3) | (17) 2.94 ( 1.5) | (80) 2.84 ( 1.4) | .30 |
| Medication tracking software | (29) 3.24 ( 1.7) | (34) 3.44 ( 1.6) | (17) 3.29 ( 1.6) | (80) 3.34 ( 1.6) | .88 |
| Remote home monitoring | (28) 1.64 ( 1.1) | (34) 2.00 ( 1.3) | (17) 1.59 ( 1.0) | (79) 1.78 ( 1.2) | .37 |
| Video consultation | (28) 2.71 ( 1.8) | (34) 3.18 ( 1.8) | (17) 3.59 ( 1.6) | (79) 3.10 ( 1.8) | .28 |
| Connected medical devices | (29) 3.03 ( 1.8) | (34) 3.29 ( 1.5) | (17) 2.71 ( 1.7) | (80) 3.08 ( 1.6) | .48 |
| Virtual reality | (29) 2.90 ( 1.4) | (34) 3.59 ( 1.4) | (17) 4.53 ( 1.1) | (80) 3.54 ( 1.4) | **<.001** |
| Robotics | (28) 1.46 ( 1.0) | (33) 1.55 ( 1.0) | (17) 1.35 ( 0.7) | (78) 1.47 ( 0.9) | .79 |
| Clinical databases | (29) 3.21 ( 1.5) | (34) 4.06 ( 0.9) | (17) 4.59 ( 1.0) | (80) 3.86 ( 1.3) | **<.001** |
| Artificial intelligence | (28) 1.89 ( 1.2) | (34) 2.53 ( 1.6) | (16) 2.50 ( 1.4) | (78) 2.29 ( 1.5) | .19 |

Pairwise comparisons (Tukey post hoc tests):

EMRs: Group 1 vs. Group 2: P =.008; Group 1 vs. Group 3: P = .179; Group 2 vs. Group 3: P = .725

Virtual Reality: Group 1 vs. Group 2: P = .104; Group 1 vs. Group 3: P < .001; Group 2 vs. Group 3: P = 0.050

Databases: Group 1 vs. Group 2:P = .014; Group 1 vs. Group 3: P < .001; Group 2 vs. Group 3: P = .284

Table 4. Current Training Coverage by Group and Mean Comparisons (Mean ± SD)

| Technology, (n) mean (SD) | Group 1 (n=58) | Group 2 (n=55) | Group 3 (n=23) | Total (N=136) | pP value |
| --- | --- | --- | --- | --- | --- |
| General technology use | (27)1.89 ( 1.2) | (31) 1.84 ( 1.1) | (17) 1.65 ( 1.2)7 | (75) 1.81 ( 1.2) | .79 |
| Specialized websites | (27)2.41 ( 1.1)2 | (31) 2.90 ( 1.1) | (17) 2.53 ( 1.1) | (75) 2.64 ( 1.1) | .21 |
| Mobile applications | (27)1.41 ( 1.0) | (28)1.21 ( 0.8) | (17) 1.12 ( 0.3) | (72) 1.26 ( 0.8) | .49 |
| Electronic medical records (EMRs) | (27)1.85 ( 1.2) | (29) 2.21 ( 1.2) | (16) 1.88 ( 1.1) | (72) 2.00 ( 1.8) | .48 |
| Scheduling software¹ | (27)1.74 ( 1.1) | (29) 1.59 ( 0.9) | (16) 1.13 ( 0.34 | (72) 1.54 ( 0.9) | **.007^(1)^** |
| Nursing care plan software | (26) 2.15 ( 1.1) | (29) 1.90 ( 0.9) | (16) 2.19 ( 1.22 | (71) 2.06 ( 1.0) | .56 |
| Medication tracking software | (27)2.26 ( 1.1) | (27)2.44 ( 1.2) | (16) 2.13 ( 1.15 | (70) 2.30 ( 1.2) | .67 |
| Remote home monitoring | (26) 1.35 ( 0.6) | (27)1.44 ( 0.8) | (14) 1.50 ( 1.16 | (67) 1.42 ( 0.8) | .84 |
| Video consultation | (25)1.80 ( 1.2) | (28)1.89 ( 1.1) | (16) 1.88 ( 1.02 | (69) 1.86 ( 1.1) | .95 |
| Connected medical devices | (26) 2.15 ( 1.4) | (27)2.04 ( 1.1) | (16) 1.94 ( 1.06 | (69) 2.06 ( 1.2) | .85 |
| Virtual reality | (26) 2.38 ( 1.3) | (27)3.00 ( 1.2)8 | (16) 3.25 ( 1.13 | (69) 2.83 ( 1.3) | .06 |
| Robotics | (24) 1.33 ( 0.80 | (27)1.30 ( 0.7) | (14 ) 1.14 ( 0.36 | (65) 1.28 ( 0.6) | .68 |
| Clinical databases | (26) 2.54 ( 1.1) | (30) 2.97 ( 1.0) 6 | (16) 3.69 ( 1.08 | (72 ) 2.97 ( 1.1) | **.004** |
| Artificial intelligence | (25)1.36 ( 0.9) | (28)1.46 ( 0.7) | (15) 1.47 ( 0.92 | (68) 1.43 ( 0.8) | .88 |

Pairwise comparisons:

Clinical databases: Group 1 vs. Group 2: P= 0.291; Group 1 vs. Group 3: P = .003; Group 2 vs. Group 3:Pp = .077

Scheduling software²: Group 1 vs. Group 2: P= 0.842; Group 1 vs. Group 3: P= .039; Group 2 vs. Group 3:P = .040

¹ p-value calculated using Welch's ANOVA due to unequal variances

² Pairwise comparisons performed using Games-Howell test

Table 5. Level of Expertise Required to Effectively Use Each Technology or Digital Tool in a Professional Context (Mean ± SD)

| Level of Expertise, (n) mean (SD) | Group 1 (n=58) | Group 2 (n=55) | Groupe 3 (n=23) | Total (N=136) | P value |
| --- | --- | --- | --- | --- | --- |
| General technology use | (27)1.52 ( 0.6) | (31) 1.32 ( 0.5) | (17) 1.59 ( 0.7) | (75) 1.45 ( 0.6) | .29 |
| Specialized websites | (27)1.93 ( 0.8) | (31) 2.13 ( 0.9) | (17) 2.41 ( 0.9) | (75) 2.12 ( 0.8) | .18 |
| Mobile applications | (26) 1.73 ( 0.8) | (26) 1.81 ( 0.8) | (17) 1.53 ( 0.8) | (69) 1.71 ( 0.8) | .56 |
| Electronic medical records (EMRs) | (26) 2.62 ( 0.9) | (29) 2.93 ( 0.6) | (17) 2.76 ( 0. 7) | (72) 2.78 ( 0.8) | .32 |
| Scheduling software | (27)2.07 ( 0.7) | (28)2.14 ( 0.7  ) | (16) 2.13 ( 0.72 | (71) 2.11 ( 0.7) | .93 |
| Nursing care plan software | (26) 2.65 ( 0.9) | (28)2.57 ( 0.7) | (17) 2.88 ( 0.78 | (71) 2.68 ( 0. 8) | .44 |
| Medication tracking software | (27)2.74 ( 0.8)6 | (27)2.67 ( 0.7) | (17) 2.82 ( 0.81 | (71) 2.73 ( 0.8) | .80 |
| Remote home monitoring | (25)2.68 ( 0.8) | (26) 2.96 ( 0.7) | (16) 2.75 ( 0.58 | (67 ) 2.81 ( 0.7) | .36 |
| Video consultation | (24) 2.04 ( 0.9) | (28)2.36 ( 0.9) | (17) 2.35 ( 0.86 | (69) 2.25 ( 0.9) | .38 |
| Connected medical devices | (26) 2.62 ( 0.8) | (27)2.59 ( 0.7) | (17) 2.71 ( 0.69 | (70) 2.63 ( 0.7) | .88 |
| Virtual reality | (26) 2.12 ( 0.7) | (27)2.63 ( 0.9) | (17) 2.35 ( 0.79 | (70) 2.37 ( 0.8) | .07 |
| Robotics | (24) 2.67 ( 0.9) | (26) 2.88 ( 0.9) | (16) 2.06 ( 0.85 | (66) 2.61 ( 0.9) | **.02** |
| Clinical databases | (26) 2.88 ( 0.7) | (30) 2.70 ( 0.8) | (17) 2.82 ( 0.64 | (73) 2.79 ( 0.7) | .65 |
| Artificial intelligence | (25)2.20 ( 0.9) | (25)2.28 ( 0.7) | (17) 2.12 ( 0.86 | (67) 2.21 ( 0.8) | .82 |

Pairwise comparisons (Tukey post hoc tests):

Robotics: Group 1 vs. Group 2: P = 0.659; Group 1 vs. Group 3: P = .093; Group 2 vs. Group 3: P = .013
