# Supplementary material for: Perceptions and Intentions of Nursing Students Regarding Digital Health: Cross-Sectional Study
Source: JMIR Nurs. 2026 Mar 5;9:e77051. doi: 10.2196/77051 (PMC12978967; doi:10.2196/77051)
Supplement: Multimedia Appendix 2 [file nursing-v9-e77051-s002.docx]

Ce questionnaire a pour objectif d’évaluer le niveau de préparation des étudiants en sciences infirmières à utiliser des technologies de santé numérique et de recueillir des informations sur leurs perceptions et besoins de formation dans ce domaine.

Ainsi, vos réponses nous permettront de mieux comprendre votre contexte et votre expérience. Toutes vos réponses resteront anonymes et confidentielles. Veuillez répondre aux questions le plus sincèrement possible. Si une question ne s'applique pas à vous ou si vous préférez ne pas répondre, vous avez cette option à votre disposition. Merci de votre collaboration !

## Section 1 : Votre profil

Q1.1. En quelle année/programme de formation êtes-vous présentement ?

- Microprogramme de 1er cycle d’intégration en sciences infirmières
- B. Sc. inf., DEC-bac, 1^re^ année
- B. Sc. Inf., DEC-bac, 2e année ou plus
- B. Sc. inf., 1re année
- B. Sc. inf., 2e année
- B. Sc. inf., 3e année ou plus
- Préfère ne pas répondre

Q1.2. Si applicable, précisez vos études post-secondaires antérieures (ex., DEC en sciences infirmières, Bac en psychologie, Certificat en informatique appliquée). ____________________________________________

Q1.3. Dans quelles langues êtes-vous à l'aise d’interagir avec un site web, un logiciel ou un ordinateur? (Vous pouvez cocher plusieurs options) :

- Français
- Anglais
- Autre : ___
- Préfère ne pas répondre

Q1.4. Dans votre contexte quotidien (professionnel et/ou personnel), quels appareils utilisez-vous sur une base régulière? (Vous pouvez cocher plusieurs options).

- Téléphone intelligent
- Tablette numérique
- Ordinateur portable
- Ordinateur de bureau
- Autre : __________

Q1.5. Avez-vous une expérience professionnelle antérieure dans le domaine de la santé?

- Oui, avec usage d'outils numériques (p.ex., dossier clinique informatisé, dossier médical électronique, télésurveillance à domicile, téléconsultation)
- Oui, sans usage notable d'outils numériques
- Non

Q1.6. Quel âge avez-vous ?

- 18-25 ans
- 26-35 ans
- 36-45 ans
- 46-55 ans
- 56-65 ans
- 66 ans ou plus
- Préfère ne pas répondre

Q1.7. Êtes-vous :

- Une femme
- Un homme
- Autre : ____
- Préfère ne pas répondre

Q1.8. Dans quel secteur prévoyez-vous œuvrer à la suite de l’obtention de votre diplôme en soins infirmiers?

- Secteur communautaire de la santé (Organismes communautaires, associations à but non lucratif, etc...)
- Secteur privé de la santé (clinique privée, etc…)
- Secteur publique provincial de la santé (CLSC, CHSLD, etc…)
- Secteur public fédéral de la santé (Santé Canada, etc…)
- Secteur académique (Université, Cégep, etc…)
- Travailleur.se autonome (infirmière indépendante, consultante en soins de santé, etc...)
- Autre secteur
- Je suis indécise ou indécis ou je n’ai pas encore décidé

Q1.9. Dans quel milieu professionnel avez-vous travaillé ou travaillez-vous actuellement?

- Secteur communautaire de la santé (Organismes communautaires, associations à but non lucratif, etc...)
- Secteur privé de la santé (clinique privée, etc…)
- Secteur publique provincial de la santé (CLSC, CHSLD, etc…)
- Secteur public fédéral de la santé (Santé Canada, etc…)
- Secteur académique (Université, Cégep, etc…)
- Travailleur.se autonome (infirmière indépendante, consultante en soins de santé, etc...)
- Je n’ai pas encore travaillé en tant qu'infirmière
- Autre secteur
- Préfère ne pas répondre

## Section 2 : Vos compétences technologiques et votre formation en santé numérique

La prochaine section se concentre sur vos compétences technologiques et votre formation en matière de technologies de l'information (TI) appliquées au domaine de la santé. À l'ère actuelle, il est essentiel pour les infirmières et infirmiers cliniciens de maîtriser ces compétences afin d’offrir des soins de qualité et s'adapter à l'évolution rapide des outils numériques dans le domaine de la santé. Veuillez évaluer à travers les questions ci-dessous votre maîtrise de chaque outil numérique, ainsi que la couverture de ces sujets dans votre programme de formation. Vos réponses aideront à évaluer où se situent les besoins de formation et d'amélioration.

**Q2.1. Comment évaluez-vous votre maîtrise et votre formation concernant les technologies et outils numériques suivants dans le contexte des soins infirmiers?** Pour chaque technologie ou outil, veuillez indiquer :

1. Votre niveau de **maîtrise** actuel.
2. La **source** principale à partir de laquelle vous avez acquis cette maîtrise.
3. Comment votre programme de **formation** couvre ce sujet.
4. Le niveau d’**expertise** que vous estimez nécessaire pour utiliser efficacement cette technologie ou cet outil numérique dans le contexte professionnel.

***Exemple fictif*** : Pour la technologie "*Vidéoconférence*", vous pourriez indiquer une maîtrise "*Très élevée*" acquise principalement par "*Apprentissage personnel*" et dans le cadre d’une " Expérience professionnelle ", sentir que votre formation la couvre "*Faiblement*", et estimer qu'une "*Formation de base*" serait suffisante pour l'utiliser adéquatement.

|  | **Maîtrise** | **Source** | **Couverture actuelle dans la formation** | **Expertise requise** |
| --- | --- | --- | --- | --- |
|  | 1. Nulle; 2. Très faible; 3. Faible; 4. Modérée; 5. Élevée; 6. Très élevée | 🞎 Apprentissage personnel ou autodidacte;  🞎 Cours académiques ou formation formelle;  🞎 Stage clinique;  🞎 Expérience professionnelle clinique;  🞎 Autre : ___ | 1. Pas du tout couvert  2. Faiblement couvert  3. Modérément couvert  4. Bien couvert  5. Très bien couvert | 1. Aucune formation nécessaire;  2. Formation de base;  3. Formation fonctionnelle ou intermédiaire;  4. Formation d'expert ou spécialisée |
| Technologies générales (ex., Internet, WhatsApp, LinkedIn, Twitter, Facebook) |  |  |  |  |
| Sites web spécialisés pour soutenir mes connaissances cliniques propres aux soins infirmiers (ex., OIIQ, RNAO, INSPQ) |  |  |  |  |
| Applications mobiles liées à la santé (ex., Novi-Chek, Qalio, Daily Yoga, etc.) |  |  |  |  |
| Dossier médical électronique (DMÉ) ou dossier clinique informatisé (DCI) |  |  |  |  |
| Logiciel de gestion des horaires |  |  |  |  |
| Logiciel de gestion des plans de soins infirmiers, des plans de traitement, des plans de travail, etc. |  |  |  |  |
| Logiciel de gestion du suivi de la médication (administration du bon médicament, au bon patient, au bon moment) |  |  |  |  |
| Télésurveillance à domicile (ex., suivi à distance de l’état de santé des malades chroniques comme MPOC) |  |  |  |  |
| Téléconsultation par vidéoconférence avec des patients ou avec d’autres professionnels de la santé |  |  |  |  |
| Objets médicaux connectés (ex., tensiomètre connecté, balance connectée, pilulier intelligent, assistant virtuel) |  |  |  |  |
| Réalité virtuelle (ex., simulation virtuelle lors de la formation clinique) |  |  |  |  |
| Robots (ex., livraison d’échantillons sur les unités de soins, chirurgie, de compagnie) |  |  |  |  |
| Bases de données (CINAL, Medline, Statistique Canada, bases de données cliniques ou administratives d’un milieu hospitalier) |  |  |  |  |
| Intelligence artificielle (ex., Logiciel offrant des prédictions ou du soutien à la décision clinique utilisant l’IA) |  |  |  |  |

Q2.2 Si pertinent, quelles autres technologies ou outils numériques vous apparait essentiels à la pratique infirmière et devrait, selon-vous, être enseignés dans le cadre de votre programme de formation?

|  |
| --- |
|  |
|  |
|  |
|  |
|  |
|  |

## Section 3 : Vos perceptions en matière d’impact de la santé numérique sur la profession infirmière

Q3.1 Quel impact croyez-vous que le recours aux technologies de santé numérique a ou aura sur les dimensions suivantes de la profession infirmière?

|  | Impact très négatif | Impact négatif | Impact neutre | Impact positif | Impact très positif |
| --- | --- | --- | --- | --- | --- |
| La qualité de la relation avec les patients (relation thérapeutique) |  |  |  |  |  |
| La qualité des soins infirmiers dispensés aux patients |  |  |  |  |  |
| La qualité de vie au travail du personnel infirmier |  |  |  |  |  |
| La productivité du personnel infirmier |  |  |  |  |  |
| L’intérêt de la relève envers la profession infirmière |  |  |  |  |  |

Q3.2 Partagez en quelques mots ci-dessous votre enthousiasme ou vos préoccupations concernant l’usage de technologies numériques et ses effets actuels ou futurs sur la profession infirmière.

|  |
| --- |
|  |
|  |
|  |
|  |
|  |
|  |

## Section 4 : Vos commentaires additionnels

Q4.1 Veuillez indiquer ci-dessous tout autre commentaire nous permettant de mieux comprendre **votre expérience et vos attitudes envers les technologies de santé numérique** à titre d’étudiante ou d’étudiant en sciences infirmières.

|  |
| --- |
|  |
|  |
|  |
|  |
|  |
|  |

**Merci pour votre participation !**

Votre contribution est essentielle pour mieux comprendre l'intégration des technologies de santé numérique dans la formation en soins infirmiers à l’Université de Montréal. Si vous souhaitez recevoir un résumé des résultats, veuillez fournir une adresse courriel : ***[Espace pour saisir l'adresse e-mail]***
